# Novel insights into cardiac structure, function, perfusion, and tissue characteristics in liver cirrhosis: a magnetic resonance analysis

**DOI:** 10.1007/s00330-025-11710-1

**Published:** 2025-06-05

**Authors:** Jennifer Erley, Lieda Naimi, Isabel Molwitz, Destina G. Aydemir, Ersin Cavus, Kai Muellerleile, Katharina A. Riedl, Mathias Meyer, Martina R. Sterneck, Gunnar K. Lund, Stefan Blankenberg, Gerhard Adam, Enver Tahir

**Affiliations:** 1 Department of Diagnostic and Interventional Radiology and Nuclear Medicine, University Medical Center Hamburg-Eppendorf, Hamburg, Germany; 2 Department of Cardiology, University Heart and Vascular Center Hamburg Eppendorf, Hamburg, Germany; 3 Deutsches Zentrum für Herz-Kreislauf-Forschung e.V. (German Center for Cardiovascular Research), Partner Site Hamburg/Kiel/Lübeck, Lübeck, Germany; 4 Department of Liver Transplantation, University Medical Center Hamburg-Eppendorf, Hamburg, Germany; 5 Department of Medicine, Center for Internal Medicine, University Medical Center Hamburg-Eppendorf, Hamburg, Germany

**Keywords:** Liver cirrhosis, Cardiac, Cardiomyopathy, Magnetic resonance imaging

## Abstract

**Objective:**

This study analyzed the impact of liver cirrhosis on cardiac structure, function, tissue characteristics, and stress perfusion using cardiac magnetic resonance (CMR) imaging.

**Materials and methods:**

Fifty patients with liver cirrhosis and 25 matched, healthy controls received a 3-T CMR exam. Left and right ventricular (LV, RV) and atrial (LA, RA) volumes and functions were analyzed, including ejection fraction (EF), and feature tracking strain analysis. T1/T2 relaxation times and extracellular volume (ECV) were determined.

**Results:**

Patients with cirrhosis showed a higher LVEF (66.6 ± 5.8 vs. 59.6 ± 4.5%, *p* < 0.001) and RVEF (65.4 ± 6.5 vs. 55.7 ± 8.0%, *p* < 0.001). LV (86.1 ± 16.2 vs. 78.0 ± 14 mL/m^2^, *p* = 0.038) volumes were higher in patients (48.8 ± 15.5 vs. 33.2 ± 9.1 mL/mL/m^2^, *p* = 0.014). RV volumes were also higher, but not statistically significant (85.1 ± 17.6 vs. 78.7 ± 13.8 mL/m^2^, *p* = 0.116). LV (−20.1 ± 3.4 vs. −17.1 ± 2.1%, *p* < 0.001) and RV global longitudinal strain (−25.8 ± 5.5 vs. −21.2 ± 5.4%, *p* = 0.001) were enhanced, similar to LV radial/circumferential strain. The Child-Pugh class and the model of end-stage liver disease (MELD) score were associated with an increase in ECV of 4.8 [1.8 to 7.8]% (*p* = 0.003) and 0.5 [0.2 to 0.8]% (*p* < 0.001). MELD was also associated with end-diastolic volumes (0.9 [0.1 to 1.7] mL/m^2^, *p* = 0.030 for the LV and 1.0 [0.1 to 1.9] mL/m^2^, *p* = 0.029 for the RV). No patient showed a perfusion deficit, while 20% showed non-ischemic late gadolinium enhancement (LGE).

**Conclusion:**

Liver cirrhosis patients showed cardiac dilatation and increased ventricular function compared to controls. Non-ischemic LGE was present in 20% of patients. Disease severity scores were linked to an increased ECV and biventricular volumes.

**Key Points:**

***Question*** *The effect of liver cirrhosis on cardiac structure, function, perfusion, and tissue characteristics has not been sufficiently investigated using cardiac MR.*

***Findings*** *Subjects with liver cirrhosis showed cardiac dilation, increased biventricular function compared to controls, non-ischemic LGE, and an association between disease severity and the extracellular volume.*

***Clinical relevance*** *Liver cirrhosis results in cardiac changes characterized by MR, predominantly dilatation and hypercontractility, as well as signs of myocardial fibrosis and elevated extracellular volume with increasing disease severity.*

**Graphical Abstract:**

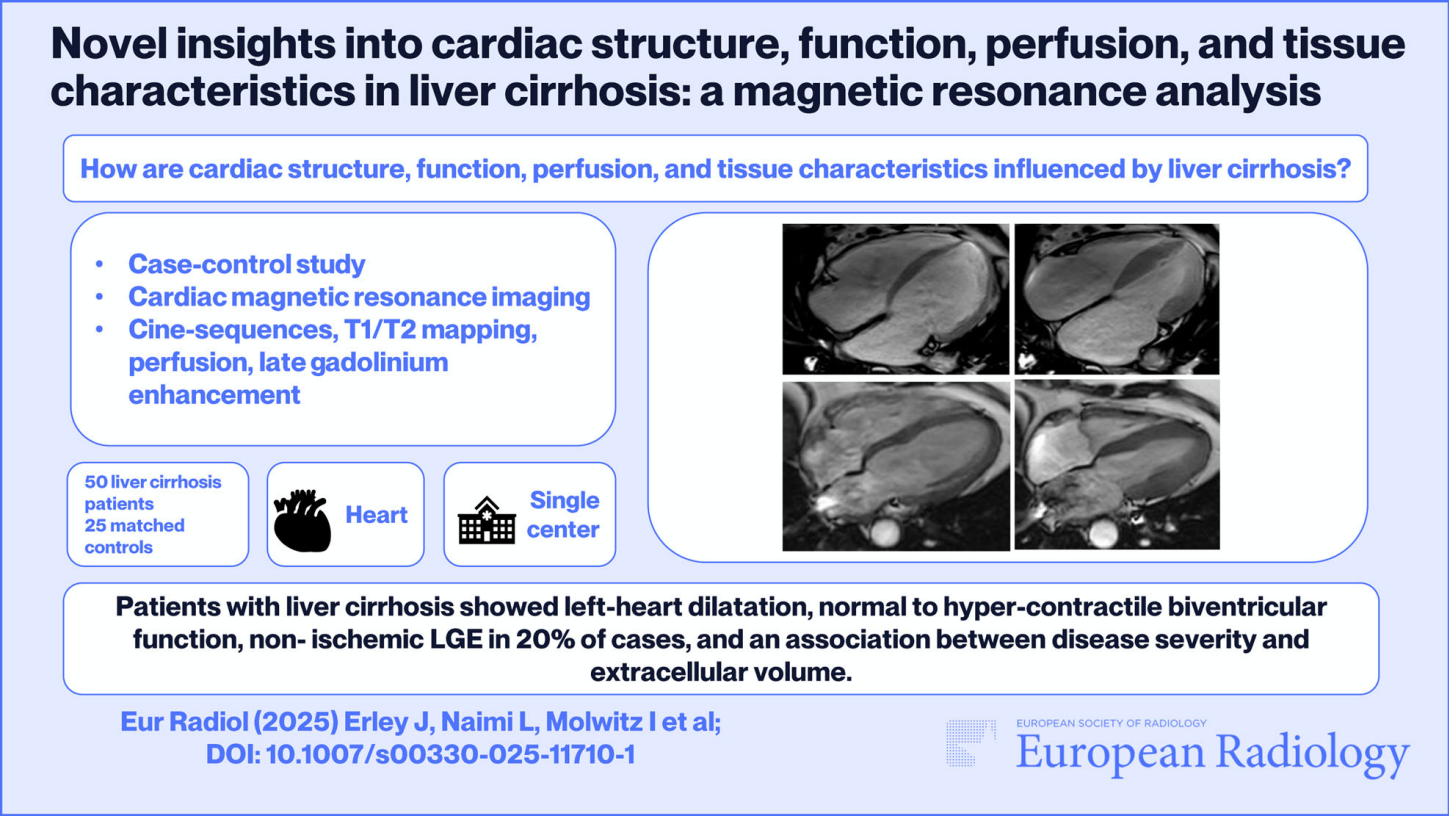

## Introduction

Despite advances in the diagnosis and management of liver cirrhosis, liver transplantation remains the only curative therapeutic option for liver cirrhosis [1]. Due to the improvement in surgical techniques, immunosuppressive medication, and management of infectious diseases, cardiovascular diseases are nowadays an advancing cause of death after liver transplantation [2, 3].

Two different factors could explain this phenomenon. First, patients with hepatic pathologies have a high cardiovascular risk profile and are likely to suffer from cardiovascular diseases, such as coronary artery disease [4]. Moreover, recent research unveiled a direct connection between liver dysfunction and cardiac damage, subsumed under the term “cirrhotic cardiomyopathy.” According to the 2019 published “cirrhotic cardiomyopathy consortium” criteria, cirrhotic cardiomyopathy is defined as a combination of systolic and diastolic dysfunction [5]. The systolic function can be determined using the ejection fraction (EF), as well as myocardial strain (which quantifies the deformation of myocardial fibers from end-diastole to end-systole) in cardiovascular magnetic resonance (CMR) and echocardiography. The criteria for systolic dysfunction in cirrhotic cardiomyopathy are met at a left ventricular (LV) EF of 50% or less, and a global longitudinal strain (GLS) of higher than −18% [5].

CMR is the gold standard for the characterization of cardiac morphology and function [6]. However, only a few studies investigated CMR imaging features in subjects with liver cirrhosis, primarily focusing on the LV. We hypothesized that subjects with liver cirrhosis awaiting transplantation would show decreased biventricular function, as well as changes in ventricular and atrial structural CMR parameters and tissue characteristics compared to age- and sex-matched controls. Furthermore, we hypothesized an association between disease severity (assessed using the Child-Pugh and the model for end-stage liver disease (MELD) score) with the mentioned CMR parameters. Moreover, we hypothesized that myocardial ischemia would be prevalent in subjects with liver cirrhosis due to a high cardiovascular risk profile.

## Materials and methods

This prospective, cross-sectional study was approved by the local ethics committee and was conducted according to the Declaration of Helsinki and in compliance with local ethical guidelines. Written consent was obtained from all study participants. This manuscript follows the STROBE guidelines [7].

### Study population

Patients with liver cirrhosis, listed for liver transplantation and regularly visiting the liver transplantation outpatient clinic of the University Medical Center Hamburg-Eppendorf, were asked to participate in this study from July 2022 to February 2024. A control group of healthy participants (matched according to age, gender, and body mass index), who underwent contrast-enhanced CMR without stress perfusion imaging for other causes, was used as a comparison. Exclusion criteria were the inability to provide informed consent, general CMR contraindications, age under 18 years, pregnancy, claustrophobia, acute heart failure class III or above (according to the New York Heart Association), cardiac arrhythmia, and a history of cardiac diseases or interventions. Specific contraindications for the administration of intravenous contrast agent were renal failure with a glomerular filtration rate of < 30 mL/min/1.73 m^2^ and a previous allergic reaction. Contraindications for the intravenous administration of regadenoson were an atrioventricular block or sinus node dysfunction, known hypersensitivity to regadenoson, systolic blood pressure of less than 90 mmHg, and constrictive airway disease.

### CMR imaging

CMR was performed on a 3-T scanner. Details of the scanning protocol can be found in the Supplementary Table 1. In patients who did not show any contraindications and who consented to the injection of regadenoson and contrast agent, stress perfusion images were acquired after injection of 400 μg regadenoson (GE Healthcare), during the first pass of 0.05 mmol/kg gadoterate meglumine (Dotarem, Guerbet). Ten minutes after applying another 0.10 mmol/kg of gadoterate meglumine, LGE images were acquired.

### CMR post-processing

Data were analyzed using commercially available software (cvi 42, Circle Cardiovascular Imaging Inc.) by two independent investigators (L.N. and J.E.) with 2 and 6 years of experience. Patients with an ischemic scar on LGE imaging, which was restricted to a coronary artery territory and therefore indicative of myocardial infarction, were excluded from further analyses, as the scar would bias further measurements to investigate the effect of liver cirrhosis. Epi- and endocardial contours of the left and right ventricle (RV) were semi-automatically contoured in the cine-CMR short-axis stack at end-diastole and end-systole to compute ventricular end-diastolic, systolic, stroke volumes, EF, and LV end-diastolic mass. Contours were manually corrected if needed, and care was taken to exclude trabeculae and papillary muscles from the contours. Right and left atrial (RA and LA) endocardial contours were drawn in cine-CMR long-axes to calculate LA and RA volumes (more information provided in the supplementary material). Volumetric results were indexed to the body surface area (EDVi: end-diastolic volume index, ESVi: end-systolic volume index, SVi: stroke volume index). Biventricular strain was assessed using “Feature Tracking” (“tissue tracking” module in cvi42, Circle Vascular Imaging). GLS was computed in the long-axis views while the short-axis cine-stack was used to calculate circumferential (GCS) and radial strain (GRS). As the myocardial fibers in the longitudinal and circumferential orientation shorten during the myocardial contraction, while the radial fibers thicken, longitudinal and circumferential strain have negative values, and radial strain values are positive. Native T1 and T2 relaxation times were manually determined at the mid-anteroseptal segment (segment VIII according to the American Heart Association segment model) [8], as septal measurements were shown to be most robust [9, 10]. The extracellular volume (ECV) was derived from the native and post-contrast T1 relaxation of the midventricular myocardium and the blood pool, as well as the patients’ hematocrit, according to the commonly used formula [9]. LGE images were visually analyzed by a Level III certified cardiac imaging expert (E.T.).

### Collection of clinical and laboratory data

In cirrhotic patients, information on the underlying liver disease resulting in the need for transplantation, known cardiovascular diseases and interventions, cardiovascular risk factors (nicotine abuse, hyperlipidemia, obesity, diabetes, arterial hypertension, chronic kidney injury), and medication (ß-blockers, ACE-inhibitors, AT1-antagonists, lipid-lowering medication, ASS, diuretics, calcium-antagonist, nitrates, and anticoagulant medication) was gathered from a questionnaire and the hospital-own electronic medical database on the day of the CMR exam. Laboratory analyses were performed on the day of the CMR, including markers of hepatobiliary disease progression (bilirubin, albumin, aspartate aminotransferase (AST) and alanine aminotransferase (ALT)), hematocrit, and cardiac markers (troponin, NTproBNP).

### Statistical analysis

Statistical analyses were conducted using SPSS for Mac (version 29.0, IBM SPSS Statistics). All measurements were assessed for normality using the Shapiro–Wilk test. Normally distributed data are expressed as mean ± standard deviation, and non-normally distributed data are described using median [interquartile range]. Differences between patients with liver cirrhosis and controls were assessed using the chi-square test, Mann–Whitney *U*-test, and Student’s *t*-test for independent variables, respectively. The association between the Child-Pugh/MELD score with CMR parameters was assessed using linear regression models after assumption testing (considering the normal distribution of the model residuals), including sex, age, and BMI.

## Results

54 liver cirrhosis patients awaiting liver transplantation prospectively underwent CMR as part of this study from July 2022 to February 2024. One patient was excluded due to nondiagnostic images, and three patients were excluded from further analyses due to ischemic LGE, leaving 50 patients for the final analysis. Of these, 44 (88%) agreed to and received intravenous contrast agent and regadenoson stress testing. No subject reported a known cardiac disease or a previous cardiac intervention. The patients were compared to 25 healthy controls. Due to motion artifacts, four patients needed to be excluded from the RA volume analysis, one patient from the LA analysis, four patients from mapping, one patient from the LV strain analysis, and three from the RV strain analysis. Table 1 shows the demographic information of the patients and the control group. There were no differences between patients with liver cirrhosis and controls regarding sex distribution, age, body surface area, and body mass index. Most patients with liver cirrhosis were categorized as Child-Pugh class B (57%), and the average MELD score was 14 ± 6 points. The most common cause of liver cirrhosis was alcohol-related liver disease (24%). The most commonly used medications were beta-blockers (46% of patients, of which 98% received unselective beta-blockers due to portal hypertension) and diuretics (54% of patients, all due to ascites). Of all the assessed cardiovascular risk factors, only the association between diabetes and RVGCS was significant in patients (Supplementary Table 2).

**Table 1:** Demographic characteristics of the study population

	Liver cirrhosis (*n* = 50)	Controls (*n* = 25)	*p*-value	Mean difference	95% CI of the difference
Clinical parameters					
Age, years	53 ± 12	56 ± 13	0.532	−3	−8 to −4
Male sex, *n* (%)	27 (54)	11 (44)	0.341		
Body surface area, m²	1.9 ± 0.2	1.9 ± 0.2	0.748	0	−0.1 to 0.1
Body mass index, kg/m^2^	26.1 ± 4.8	26.8 ± 4.6	0.507	−0.7	−3.1 to 1.5
MELD score, points	14 ± 6	/			
Child-Pugh class, *n* (%)	16 (35) A; 26 (56) B; 4 (9) C	/			
Blood pressure at rest (mmHg)	120 ± 15/69 ± 11	/			
Heart rate at rest (bpm)	66 ± 15	/			
Blood pressure during stress (mmHg)	121 ± 18	/			
Heart rate during stress (bpm)	73 ± 19	/			
Cardiovascular risk factors					
Arterial hypertension, *n* (%)	7 (14)	/			
Obesity, *n* (%)	10 (20)	/			
Smoking, *n* (%)	3 (6)	/			
Diabetes, *n* (%)	10 (20)	/			
Hyperlipidemia, *n* (%)	4 (8)	/			
Cardiovascular medication					
Beta-blockers, *n* (%)	23 (46)	/			
ACE-inhibitors, *n* (%)	3 (6)	/			
AT1 receptor antagonists, *n* (%)	1 (2)	/			
Statins, *n* (%)	1 (2)	/			
Diuretics, *n* (%)	27 (54)				
Laboratory analysis					
Hemoglobin, g/dL (ns)	12.1 [10.7 to 13.6]	/			
Hematocrit, %	36.4 ± 5.1	/			
Albumin, g/L	32.3 ± 6.9	/			
Bilirubin, mg/dL (ns)	2.4 [1.2 to 3.8]	/			
Creatinine, mg/dL (ns)	0.8 [0.7 to 1.0]	/			
ASAT, U/l (ns)	48.5 [34.5 to 77.5]	/			
ALAT, U/l (ns)	31.5 [24.0 to 83.8]	/			
Troponin, pg/mL	3.8 ± 2.7	/			
NTproBNP, ng/L (ns)	93.5 [52.5 to 207.3]	/			

Numbers are mean ± standard deviation (SD) or median [interquartile range] for continuous and *n* (%) for categorical data. The blood pressure and heart rate during stress were measured after the regadenoson administration before acquisition of the stress perfusion images

*CI* confidence interval, *MELD* model of end-stage liver disease, *bpm* beats per minute, *ACE* angiotensin-converting enzyme, *AT1* angiotensin type 1, *ASAT* aspartate aminotransferase, *ALAT* alanine aminotransferase, *NTproBNP* N-terminal pro-B-type natriuretic peptide

36% of cirrhotic patients showed valvular diseases on CMR: mitral valve insufficiency (10%), tricuspid valve insufficiency (TI) (6%), mitral and TI (10%), aortic valve insufficiency (6%), aortic and TI (2%), and aortic and mitral valve insufficiency (2%). In patients with TI, RVESVi was higher (B = 6.8 mL/m^2^ [95% confidence interval 0.3 to 13.3], *p* = 0.039, median RVESVi of 27.9 mL/m^2^ [interquartile range 22.6 to 31.3] without and 29.5 mL/m^2^ [25.7 to 36.7] with TI), RVGRS was lower (−6.3% [−11.4 to −1.3], *p* = 0.016, mean of 21.6% ± 6.8 without and 17.6% ± 4.6 with TI), and RVGCS was higher (B = 3.6 [1.2 to 6.0], *p* = 0.005, −9.8% ± 2.9 with and −12.3% ± 3.1 without TI). Other than that, there were no significant differences in CMR parameters between the patients with and without valve deficiencies.

### CMR parameters in patients with liver cirrhosis compared to healthy controls

Table 2 displays the differences in structural and functional CMR parameters between patients with liver cirrhosis and healthy controls. Liver cirrhosis patients showed a higher LV and RV function compared to controls. This is reflected by the higher LVEF (66.6 ± 5.8 vs. 59.6 ± 4.5%, *p* < 0.001) and RVEF (66.2 [62.7 to 68.8] vs. 57.0 [51.0 to 60.5]%, *p* < 0.001), as well as a significantly lower LVGLS (−19.9 [−23.0 to −17.2] vs. −17.0 [−18.5 to −16.0]%, *p* < 0.001) and RVGLS (−25.8 ± 5.4 vs. −21.2 ± 5.4%, *p* < 0.001). This difference remained significant after excluding subjects with TI (*p* = 0.020). Similarly, LVGCS (−20.9 ± 3.1 vs. −17.4 ± 2.2%, *p* < 0.001) was significantly lower in cirrhosis patients, and LVGRS was higher (45.8 ± 10.6 vs. 31.4 ± 10.3%, *p* < 0.001). Differences in RVGCS and RVGRS were not statistically significant to the controls, also not after excluding subjects with TI (*p* = 0.061). LVEDVi and RVEDVi were higher in patients with liver cirrhosis, but only the LVEDVi was statistically significantly different from the control group (86.1 ± 16.2 vs. 78.0 ± 14.6 mL/m^2^, *p* = 0.038). LVESVi and RVESVi were lower in subjects with liver cirrhosis, resulting in a significantly higher SVi (LVSVi: 57.7 ± 11.5 vs. 46.3 ± 7.9 mL/m^2^, *p* < 0.001 and RVSVi: 55.2 ± 13.1 vs. 43.6 ± 9.1 mL/m^2^, *p* < 0.001). The difference in RVESVi increased after excluding subjects with TI (*p* = 0.004).

**Table 2:** Structural and functional CMR parameters in patients with liver cirrhosis and healthy controls

CMR-parameter	Liver cirrhosis	Controls	*p*-value	Mean or median difference	95% CI of the difference
LVEF (%)	66.6 (5.8)	59.6 (4.5)	**< 0.001**	**7.0**	**4.3 to 9.7**
LVEDMi (g/m^2^)	51.9 [46.8 to 58.8]	53.0 [48.7 to 57.0]	0.728	−1.1	−4.8 to 3.2
LVEDVi (mL/m^2^)	86.1 (16.2)	78.0 (14.6)	**0.038**	**8.1**	**0.5 to 15.8**
LVESVi (mL/m^2^)	28.3 [23.0 to 33.9]	31.1 [26.1 to 37.2]	0.113	−2.8	−0.8 to 6.5
LVSVi (mL/m^2^)	57.7 (11.5)	46.3 (7.9)	**< 0.001**	**11.4**	**6.2 to 16.4**
RVEF (%)	66.2 [62.7 to 68.8]	57.0 [51.0 to 60.5]	**< 0.001**	**9.2**	**5.8 to 12.1**
RVEDVi (mL/m^2^)	85.1 (17.6)	78.7 (13.8)	0.116	6.4	1.6 to 14.5
RVESVi (mL/m^2^)	28.5 [23.2 to 31.4]	35.9 [28.2 to 38.1]	**0.007**	**−7.4**	**−9.3 to −1.9**
RVSVi (mL/m^2^)	55.2 (13.1)	43.6 (9.1)	**< 0.001**	**11.6**	**5.8 to 17.5**
LAEDVi (mL/m^2^)	18.6 [12.2 to 26.9]	12.8 [10.5 to 16.3]	**0.014**	**5.8**	**0.9 to 8.9**
LAESVi (mL/m^2^)	49.3 [35.1 to 62.0]	31.3 [26.4 to 37.0]	**< 0.001**	**18.0**	**8.3 to 22.5**
RAEDVi (mL/m^2^)	21.1 [16.2 to 29.7]	19.7 [15.2 to 24.1]	0.373	1.4	−2.3 to 5.6
RAESVi (mL/m^2^)	42.9 (14.5)	38.7 (15.1)	0.258	4.2	0.3 to 11.5
Native T2 relaxation time (ms)	48.3 (4.2)	47.2 (2.6)	0.215	1.2	−0.7 to 3.1
Native T1 relaxation time (ms)	1232.5 (49.9)	1237.8 (37.6)	0.651	−5.3	−28.4 to 17.9
ECV (%)	27.0 [25.8 to 31.0]	27.0 [22.9 to 29.0]	0.099	0	−4.5 to 0.0
LVGLS (%)	−19.9 [−23.0 to −17.2]	−17.0 [−18.5 to −16.0]	**< 0.001**	**−2.9**	**−4.5 to −1.4**
LVGRS (%)	45.8 (10.6)	31.4 (10.3)	**< 0.001**	**14.4**	**9.3 to 19.6**
LVGCS (%)	−20.9 (3.1)	−17.4 (2.2)	**< 0.001**	**−3.5**	**−4.9 to −2.1**
RVGLS (%)	−25.8 (5.4)	−21.2 (5.4)	**0.001**	**−4.6**	**−7.4 to −1.9**
RVGRS (%)	20.9 (6.6)	18.7 (7.4)	0.215	2.2	−1.3 to 5.8
RVGCS (%)	−11.9 (3.2)	−10.5 (3.4)	0.111	−1.4	−3.1 to 0.3

Numbers are mean ± SD or median (interquartile range)

*CI* confidence interval, *LV* left ventricle, *RV* right ventricle, *LA* left atrium, *RA* right atrium, *EF* ejection fraction, *EDMi* end-diastolic mass index, *EDVi* end-diastolic volume index, *ESVi* end-systolic volume index, *SVi* stroke volume index, *ECV* extracellular volume, *GLS* global longitudinal strain, *GRS* global radial strain, *GCS* global circumferential strain

The bold values indicate if the differences between subjects with liver cirrhosis and controls are statistically significant

**Fig. 1:**
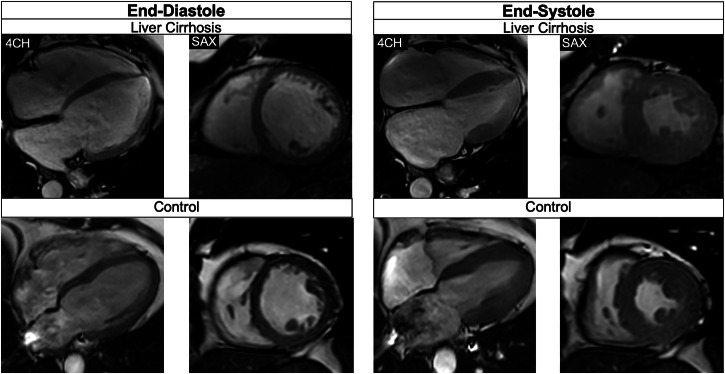
Balanced steady-state free precession cine-images at end-diastole and end-systole (4-chamber (4CH) and midventricular short axis (SAX) views) of a 51-year-old male patient with liver cirrhosis due to primary sclerosing cholangitis (MELD score: 16 points, Child-Pugh class B) and of a 53-year-old male control. The patient showed a higher LVEDVi (108.3 versus 71.8 mL/m^2^)/RVEDVi (112.1 versus 67.5 mL/m^2^), a higher LVEF (73.0 versus 64.0%)/RVEF (60.5 versus 57.0%), and increased biventricular strain (LVGLS: −26.0%, LVGRS: 39.9%, LVGCS: −20.4%, RVGLS: −22.9%, RVGRS: 12.3%, RVGCS: −8.0%) than the age-matched control (LVGLS: −14.0%, LVGRS: 19.0%, LVGCS: −17.0%, RVGLS: −22.8%, RVGRS: 13.3%, RVGCS; −7.3%). Furthermore, the patient showed an elevated ECV (32.0%) compared to the control (22.4%)

LAESVi (49.3 [35.1 to 62.0] vs. 31.3 [26.4 to 37.0] mL/m^2^, *p* < 0.001) and LAEDVi (18.6 [12.2 to 26.9] vs. 12.8 [10.5 to 16.3] mL/m^2^, *p* = 0.014) were also higher in cirrhosis patients. RA volumes were not higher in cirrhosis patients than in controls. There were no differences in native T1/T2 relaxation times and ECV between subjects with liver cirrhosis (all within normal ranges [11]). Figure 1 portrays exemplary balanced steady-state free precession cine-images (4-chamber and short-axis view) of a liver cirrhosis patient compared to an age-matched control.

**Fig. 2:**
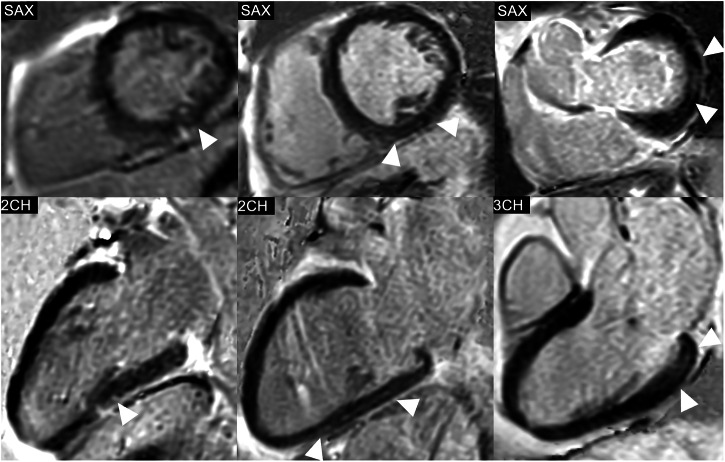
Phase-sensitive inversion recovery (PSIR) images of the left ventricle (LV) in short-axis (SAX) views and the corresponding 2-chamber (2CH) or 3-chamber (3CH) views, showing the distribution of non-ischemic late gadolinium enhancement (LGE) in three exemplary study patients. Left: Punctiform subepicardial LGE of the midventricular inferior LV in a 63-year-old male patient with cirrhosis due to primary sclerosing cholangitis (MELD score: 18 points, Child-Pugh class B). Middle: Linear mid-myocardial LGE of the inferior LV in a 54-year-old male liver cirrhosis patient with chronic Hepatitis C virus infection (MELD score: 15 points, Child-Pugh class B). Right: Linear mid-myocardial LGE of the lateral basal LV in a 57-year-old male liver cirrhosis patient with chronic Hepatitis C virus infection (MELD score: 6 points, Child-Pugh class B)

None of the patients who received stress testing demonstrated a myocardial perfusion deficit. However, 9 out of the 44 patients (20%) showed non-ischemic LGE. Figure 2 shows the patterns of LGE distribution in three exemplary patients. Most patients showed basal to midventricular LGE (6/9 patients, 66.7%), inferior (8/9 patients, 88.9%), and mid-myocardial LGE (8/9 patients, 88.9%). LGE was linear in 5 patients (55.6%), patchy in two patients (4%), and punctiform in two patients (4%).

### Association between severity of liver dysfunction with CMR parameters

**Fig. 3:**
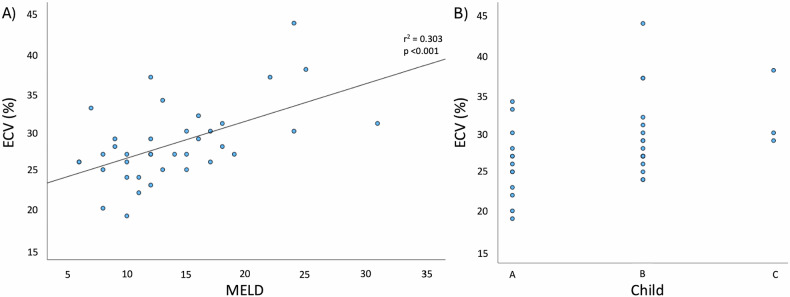
Scatter plots showing the relationship between (**A**) MELD score and (**B**) Child-Pugh class with ECV. ECV correlated significantly with MELD (*r* = 0.55, *r*^2^ = 0.303, *p* < 0.001). An increase in MELD was associated with a 0.5 [0.2 to 0.8]% higher ECV (*p* < 0.001). A higher Child-Pugh class was also associated with an increase in ECV of 4.8 [1.8 to 7.8]% (*p* = 0.003)

A higher Child-Pugh class was associated with an increase in ECV of 4.8 [1.8 to 7.8]% (*p* = 0.003), but not with changes in structural and functional CMR parameters in patients with liver cirrhosis. An increase in MELD score by one point was associated with a 0.9 [0.1 to 1.7] mL/m^2^ higher LVEDVi (*p* = 0.030), a 0.7 [0.2 to 1.3] mL/m^2^ higher LVSVi (*p* = 0.014), a 1.0 [0.1 to 1.9] mL/m^2^ higher RVEDVi (*p* = 0.029), and a 0.9 [0.2 to 1.6] mL/m^2^ higher RVSVi (*p* = 0.010). Furthermore, an increase in MELD was associated with a 0.5 [0.2 to 0.8]% higher ECV (*p* < 0.001). Figure 3 illustrates the relationship between (1) MELD and (2) Child-Pugh class with ECV.

## Discussion

This study aimed to characterize cardiac structure, function, and tissue characteristics, as well as the prevalence of myocardial ischemia in a prospective cohort of subjects with liver cirrhosis awaiting transplant. The main findings of this analysis were as follows:

1. Subjects with liver cirrhosis showed increased biventricular contractility (higher EF, lower ESVi, and higher SVi, enhanced GLS) and cardiac dilatation compared to healthy controls.
2. Child-Pugh class and MELD score were associated with the ECV as a marker of diffuse myocardial fibrosis.
3. None of the patients who received stress testing showed a reversible myocardial perfusion deficit, while 20% showed non-ischemic LGE (mostly basal to midventricular, inferior, mid-myocardial, and linear).

Patients with liver cirrhosis showed increased LV and LA volumes compared to controls in this study. RV and RA volumes were also higher in patients, but did not reach statistical significance. Moreover, contrary to our hypothesis and previous echocardiographic studies [12–14], biventricular strain and EF were normal to “hypercontractile” in patients with liver cirrhosis. Previous smaller studies by Kim et al and Isaak et al also reported an enhanced LVEF and LVGLS in patients with liver cirrhosis [15, 16], as opposed to an impaired ventricular function. The increased contractility in cirrhosis patients can be explained as a response to a hyperdynamic circulatory state in the setting of portal hypertension [5, 17]. This hyperdynamic circulation is attributed to increased levels of endocannabinoids, nitric oxide, and carbon monoxide in cirrhosis patients (due to systemic inflammation and endotoxemia), which induce systemic vasodilatation and a decrease in vascular resistance [18, 19]. This causes arterial underfilling, which triggers neurohumoral systems like the sympathetic nervous system, the renin-angiotensin-aldosterone system, and secretion of antidiuretic hormone, which in turn lead to increased cardiac output, increased contractility, and increased cardiac preload due to sodium and water retention [18, 19].

To our knowledge, this is the first CMR-based study of right ventricular structure and function, as well as atrial morphology in cirrhosis patients. Despite not being statistically significant, the differences in RV volumes between cirrhosis patients and controls can be considered clinically relevant, because the RVEDVi showed a similar relationship to the MELD score as the LVEDVi. This indicates a progressive RV dilatation with worsening liver function. Our findings highlight that cirrhotic cardiomyopathy is not confined to the LV but seems to affect all cardiac chambers, which has not been mentioned yet in diagnostic criteria and guidelines [5]. Moreover, the results of our and other CMR studies indicate that many patients with liver cirrhosis show normal-to-increased cardiac function using EF and strain imaging. These patients are not diagnosed with “cirrhotic cardiomyopathy” according to the current guidelines [5], despite showing clear differences in cardiac structure and function to healthy subjects.

In this cohort, 20% of patients showed non-ischemic LGE (mostly basal to midventricular, inferior, mid-myocardial, and linear). We also observed a relationship between disease severity scores and signs of myocardial fibrosis. The ECV was significantly associated with the Child-Pugh class and MELD score in this cohort, similar to other studies [15, 16, 20]. Moreover, 20% of patients showed LGE with a non-ischemic pattern. The association between ECV and severity of liver cirrhosis could be explained by the water retention due to the excessive neurohumoral activation in cirrhotic patients [18]. With worsening cirrhosis and portal hypertension, the expanded blood volume is redistributed from the central circulation to the splanchnic, which triggers the neurohumoral activation even further [18]. Moreover, the renin-angiotensin-aldosterone system has been described as a major contributor to reactive interstitial fibrosis [21, 22]. Despite the association between ECV and MELD/Child-Pugh class, we did not find a significantly different ECV or significantly different T1 and T2 relaxation times in cirrhosis patients compared to controls. These findings are contradictory to some other studies that reported an expanded ECV in cirrhosis patients [15, 20], but predominantly included patients with Child-Pugh class C [15]. The majority of patients in this study were Child-Pugh class B, with possibly more “compensated” patients showing normal ECV [11]. However, the interquartile range indicates a higher trend of ECV values in cirrhosis patients, which could underline the link between worsening portal hypertension and expanded ECV.

Despite preoperative cardiovascular screening, cardiovascular death is currently one of the leading causes of post-transplant mortality in cirrhosis patients [2, 3]. As shown by our and other studies, cardiovascular risk factors are often found in cirrhosis patients [23]. Accordingly, the prevalence of moderate to severe coronary artery disease in cirrhosis patients referred to coronary artery angiography was reported to be as high as 37% [4, 24]. However, none of the patients investigated here showed a reversible perfusion deficit suggestive of myocardial ischemia. In a previous study by Reddy et al, only a small number of cirrhosis patients (5%) investigated by CMR perfusion imaging showed abnormal results requiring invasive testing [25]. Thus, there might be a discrepancy between the high prevalence of coronary artery disease and the comparatively low prevalence of ischemia in cirrhosis patients. This needs to be considered when choosing a suitable preoperative cardiovascular test before transplantation, as only functionally relevant coronary artery disease is considered a contraindication for liver transplantation [2, 26].

The main limitation of this study is the single-center setting, and the limited size of patients with heterogeneous liver diseases resulting in liver cirrhosis. Due to our specialized center for autoimmune liver diseases, we included a comparatively large number of PBC/PSC patients in this study, who might be younger and of better overall health than the patients investigated in other studies. Also, this study cannot provide evidence on the previously described impaired cardiac response to pharmacological stress in cirrhosis patients, as the cine-sequence to assess structural and functional CMR parameters was not repeated after regadenoson administration. Moreover, diuretics were not suspended before the CMR exam, potentially influencing cardiac volumes. Furthermore, image quality was limited in patients who were unable to hold their breath due to ascites, and/or pleural effusions.

## Conclusion

Patients with liver cirrhosis listed for liver transplantation showed cardiac dilatation, and normal to increased biventricular function. 20% of subjects with liver cirrhosis showed non-ischemic LGE, and the ECV, as an indicator of diffuse myocardial fibrosis, was associated with the Child-Pugh class and MELD score. Despite showing a high cardiovascular risk profile, none of the investigated patients demonstrated a myocardial perfusion deficit.

## Supplementary information

ELECTRONIC SUPPLEMENTARY MATERIAL

## Abbreviations

CMR: Cardiovascular magnetic resonance
ECV: Extracellular volume
EF: Ejection fraction
ESVi: End-systolic volume index
GLS: Global longitudinal strain
LA: Left atrium
LGE: Late gadolinium enhancement
LV: Left ventricle
MELD: Model for end-stage liver disease
RA: Right atrium
RV: Right ventricle
SVi: Stroke volume index
TI: Tricuspid valve insufficiency
