# Supplementary material for: Novel insights into cardiac structure, function, perfusion, and tissue characteristics in liver cirrhosis: a magnetic resonance analysis
Source: Eur Radiol. 2025 Jun 5;35(12):8058–67. doi: 10.1007/s00330-025-11710-1 (PMC12634777; doi:10.1007/s00330-025-11710-1)
Supplement: Supplementary file 1 — ELECTRONIC SUPPLEMENTARY MATERIAL [file 330_2025_11710_MOESM1_ESM.pdf]

Novel Insights into Cardiac Structure, Function, Perfusion, and Tissue Characteristics in Liver Cirrhosis: A Magnetic Resonance Analysis

ELECTRONIC SUPPLEMENTARY MATERIAL

Methods

Manual Atrial Volume Analysis

The LA and RA contours were traced in the 4-chamber (RA and LA) and 2-chamber long-axis views (only LA) at end-diastole (before opening of the mitral valve) and at end-systole (closing of the mitral valve). The LA contour was delineated starting from the insertion point of the mitral valve, and finishing at the other mitral valve insertion point, with caution to exclude the pulmonary veins and the left atrial appendage. Likewise, the RA contour was delineated starting from the insertion point of the pulmonary valve, ending at the perpendicular insertion point, excluding the ostia of the vena cava and the right atrial appendage. The biplane area-length method (Lang et al.) [1] was automatically applied to estimate LA volumes (4-chamber area x 2-chamber area x 0.85/atrial length [2]), while the RA volumes were automatically assessed using the Simpson's method. **Supplementary Figure 1** shows a 4-chamber image at end-diastole of an exemplary study patient, including the LA contour (orange) and RA contour (blue).

Supplementary Table 1: Detailed parameters of each employed CMR sequence.

**Imaging Parameters**	**Balanced turbo field echo (BTFE) sequence**		**5 s (3 s) 3 s Modified look-locker inversion recovery sequence (MOLLI)**	**Free-breathing black-blood prepared gradient and spin-echo (GraSE) sequence**	**Ultrafast T1-weighted turbo field echo (TFE) sequence**	**Phase-sensitive inversion recovery (PSIR) sequence**	
Scan views	Short-axis stack from base to apex	2-, 3-, 4-chamber views	3 short-axis slices (basal, midventricular, apical) pre- and post-contrast	3 short-axis slices	Short-axis stack from base to apex before + after the administration of regadenoson and contrast agent	Short-axis stack from base to apex	2-, 3-, 4-chamber views
TR (m)	2.8	3.1	2.2	923	2.4	6.1	6.1
TE (ms)	1.41	1.53	0.99	n*10	1.05	3.0	3.0
Partial echo factor	/	/	0.85	/	/	/	/
Acquired voxel size (mm^3)	1.99/2.27/8.00	1.99/1.81/8.00	1.97/2.00/10.0	1.99/2.12/8.00	3.00/3.00/10.0	1.59/2.22/8.00	1.59/1.95/8.00
Reconstructed voxel size (mm^3)	0.99/0.99/8.00	0.99/0.99/8.00	1.17/1.17/10.0	0.52/0.52/8.00	1.50/1.50/10.0	0.91/0.91/8.00	0.91/0.91/8.00
Black Blood inverted delay (ms)	/	/	/	393.8	/	/	/
No. of heart phases reconstructed	30	30	Single phase	Single phase	Single phase	Single-phase	Single-phase
TFE factor	16	15	76	/	60	20	20
TSE factor	/	/	/	9	/	/	/
EPI factor	/	/	/	9	/	/	/
Echoes	1	1	1	9	1	1	1
Flip angle	45	45	20	90	20	25	25
Slice thickness (mm)	8	8	10	8	10	8	8
Reconstruction matrix	352	352	256	672	240	384	384
Acceleration	SENSE	SENSE	SENSE	SENSE	SENSE	SENSE	SENSE
P reduction (RL)	2	2	2	2.4	2	1.5	1.5
Stacks	1	1	1	1	1	1	1
Type	Parallel	Parallel	Parallel	Parallel	Parallel	Parallel	Parallel
Slices	12	1	3	3	3	10	1
Gap (mm)	2	0	12	12	7.1	2	2

Supplementary Table 2: Results of the regression analysis investigating the effect of cardiovascular risk factors on CMR structure and function in the patient cohort.

	Model 1			
CMR Parameter	Independent variables	Regression coefficient	95% confidence interval	p
**LVEF (%)**	Sex	1.9	-1.5 to 5.5	0.263
	Age	0.1	-0.1 to 0.3	0.104
	BMI	0.3	-0.1 to 0.7	0.061
	Smoking	-5.9	-13.0 to 1.2	0.099
	Hyperlipoproteinemia	-1.8	-6.7 to 3.1	0.458
	Diabetes	1.0	-5.2 to 3.2	0.630
	Arterial Hypertension	0.1	-4.9 to 5.1	0.970
**LVEDMi (g/m^2)**	Sex	-5.7	-11.4 to 0.1	0.051
	Age	-0.2	-0.4 to 0.1	0.180
	BMI	0.3	-0.3 to 0.9	0.258
	Smoking	-1.7	-13.2 to 9.9	0.771
	Hyperlipoproteinemia	2.2	-5.7 to 10.1	0.583
	Diabetes	2.7	-4.1 to 9.5	0.431
	Arterial Hypertension	0.5	-7.6 to 8.6	0.905
**LVEDVi (ml/m^2)**	Sex	-9.9	-19.5 to -0.4	**0.041**
	Age	-0.5	-0.9 to -0.1	**0.014**
	BMI	-0.1	-1.0 to 1.0	0.986
	Smoking	14.0	-5.3 to 33.3	0.150
	Hyperlipoproteinemia	0.7	-12.6 to 13.9	0.921
	Diabetes	-2.3	-13.7 to 9.1	0.692
	Arterial Hypertension	-4.5	-18.1 to 9.0	0.500
**LVESVi (ml/m^2)**	Sex	-5.2	-9.5 to -1.0	**0.017**
	Age	-0.3	-4.7 to -0.1	**0.002**
	BMI	-0.3	-0.8 to 0.1	0.119
	Smoking	6.2	-2.4 to 14.8	0.152
	Hyperlipoproteinemia	0.7	-5.2 to 6.6	0.820
	Diabetes	1.3	-3.8 to 6.4	0.616
	Arterial Hypertension	-1.4	-7.4 to 4.7	0.652
**LVSVi (ml/m^2)**	Sex	-4.2	-11.4 to 2.9	0.240
	Age	-0.2	0.5 to 0.1	0.099
	BMI	0.3	-4.6 to 1.0	0.451
	Smoking	7.9	-6.6 to 22.4	0.277
	Hyperlipoproteinemia	-0.3	-10.2 to 9.7	0.953
	Diabetes	-2.1	-10.7 to 6.5	0.622
	Arterial Hypertension	-3.2	-13.4 to 7.0	0.529
**RVEF (%)**	Sex	3.7	-0.2 to 7.6	0.062
	Age	0.2	0.1 to 0.4	**0.014**
	BMI	0.2	-0.2 to 0.6	0.369
	Smoking	-2.1	-10.0 to 5.8	0.592
	Hyperlipoproteinemia	-3.6	-9.0 to 1.8	0.187
	Diabetes	-1.3	-6.0 to 3.4	0.574
	Arterial Hypertension	0.3	-5.2 to 5.9	0.912
**RVEDVi (ml/m^2)**	Sex	-10.3	-21.0 to 0.3	0.057
	Age	-0.4	-0.8 to 0.1	0.111
	BMI	0.3	-0.8 to 1.4	0.565
	Smoking	12.5	-9.1 to 34.1	0.249
	Hyperlipoproteinemia	-0.1	-14.9 to 14.7	0.987
	Diabetes	-7.8	-20.6 to 5.0	0.225
	Arterial Hypertension	-5.6	-20.7 to 9.6	0.461
**RVESVi (ml/m^2)**	Sex	-6.9	-11.9 to -1.9	**0.008**
	Age	-0.3	-0.5 to -0.1	**0.002**
	BMI	0.1	-0.5 to 0.5	0.995
	Smoking	6.5	-3.5 to 16.6	0.196

	Hyperlipoproteinemia	2.0	-4.9 to 8.9	0.563
	Diabetes	-1.2	-7.2 to 4.7	0.674
	Arterial Hypertension	-3.0	-10.0 to 4.0	0.394
**RVSVi (ml/m²)**	Sex	-1.0	-9.3 to 7.3	0.807
	Age	-0.9	-0.4 to 0.2	0.622
	BMI	0.3	-0.6 to 1.1	0.481
	Smoking	8.4	-8.4 to 25.3	0.318
	Hyperlipoproteinemia	-7.6	-19.1 to 4.0	0.193
	Diabetes	-3.9	-13.9 to 6.1	0.436
	Arterial Hypertension	-2.0	-13.8 to 9.8	0.732
**LAEDVi (ml/m²)**	Sex	-3.8	-8.2 to 0.7	0.099
	Age	0.1	-0.1 to 0.3	0.260
	BMI	0.2	-0.3 to 0.7	0.389
	Smoking	5.3	-3.7 to 14.3	0.243
	Hyperlipoproteinemia	1.3	-4.9 to 7.5	0.667
	Diabetes	-1.2	-6.6 to 4.1	0.641
	Arterial Hypertension	-5.9	-12.3 to 0.4	0.066
**LAESVi (ml/m²)**	Sex	-6.0	-15.7 to 3.6	0.215
	Age	-0.1	-0.5 to 0.3	0.693
	BMI	0.5	-0.4 to 1.5	0.266
	Smoking	8.8	-10.7 to 28.2	0.368
	Hyperlipoproteinemia	-0.2	-13.6 to 13.1	0.971
	Diabetes	-7.2	-18.8 to 4.3	0.213
	Arterial Hypertension	-10.0	-23.7 to 3.7	0.147
**RAEDVi (ml/m²)**	Sex	-9.4	-14.7 to -4.1	**<0.001**
	Age	0.1	-0.1 to 0.3	0.351
	BMI	-0.1	-0.6 to 0.5	0.848
	Smoking	2.5	-9.8 to 14.7	0.685
	Hyperlipoproteinemia	2.1	-5.1 to 9.3	0.555
	Diabetes	-1.9	-8.2 to 4.5	0.557
	Arterial Hypertension	-6.5	-14.3 to 1.4	0.103
**RAESVi (ml/m²)**	Sex	-10.2	-19.3 to -1.0	**0.030**
	Age	-0.1	-0.5 to 0.3	0.648
	BMI	-0.6	-1.6 to 0.3	0.175
	Smoking	2.7	-18.4 to 23.9	0.794
	Hyperlipoproteinemia	5.7	-6.8 to 18.1	0.363
	Diabetes	-9.7	-20.6 to 1.3	0.081
	Arterial Hypertension	-6.6	-20.1 to 6.9	0.329
**Native T2 relaxation time (ms)**	Sex	-1.0	-3.6 to 1.6	0.446
	Age	-0.1	-0.1 to 0.1	0.433
	BMI	0.2	-0.1 to 0.5	0.076
	Smoking	3.1	-3.2 to 9.5	0.325
	Hyperlipoproteinemia	-2.4	-6.0to 1.2	0.181
	Diabetes	-0.8	-4.0 to 2.5	0.617
	Arterial Hypertension	-0.3	-4.0 to 3.3	0.860
**Native T1 relaxation time (ms)**	Sex	2.7	-28.6 to 33.9	0.863
	Age	0.5	-0.8 to 1.7	0.472
	BMI	-3.9	-7.2 to -0.6	**0.020**
	Smoking	-12.1	-87.6 to 63.3	0.747
	Hyperlipoproteinemia	-5.4	-47.9 to 37.2	0.800
	Diabetes	36.4	-2.3 to 75.1	0.065
	Arterial Hypertension	7.8	-35.6 to 51.0	0.719
**ECV (%)**	Sex	0.8	-3.3 to 4.9	0.677
	Age	0.1	-0.2 to 0.3	0.697
	BMI	-0.5	-1.0 to -0.1	**0.039**
	Smoking	1.4	-11.3 to 14.1	0.825
	Hyperlipoproteinemia	-2.8	-8.1 to 2.6	0.297
	Diabetes	0.9	-4.6 to 6.4	0.742
	Arterial Hypertension	-1.9	-7.3 to 3.5	0.480

**LVGLS (%)**	Sex	0.4	-1.8 to 2.6	0.719
	Age	0.1	-0.1 to 0.1	0.970
	BMI	-0.1	-0.3 to 0.2	0.546
	Smoking	2.1	-2.3 to 6.5	0.350
	Hyperlipoproteinemia	-1.5	-4.5 to 1.6	0.329
	Diabetes	1.7	-0.9 to 4.3	0.199
	Arterial Hypertension	0.6	-2.5 to 3.7	0.712
**LVGRS (%)**	Sex	7.7	2.0 to 13.6	**0.010**
	Age	0.4	0.1 to 0.6	**0.004**
	BMI	0.4	-0.2 to 1.0	0.208
	Smoking	-7.6	-19.3 to 4.1	0.197
	Hyperlipoproteinemia	-8.0	-16.0 to 0.1	0.053
	Diabetes	-5.0	-11.9 to 2.0	0.155
	Arterial Hypertension	-3.7	-12.0 to 4.5	0.365
**LVGCS (%)**	Sex	-2.3	-4.0 to -0.6	**0.009**
	Age	-0.1	-0.2 to -0.1	**0.002**
	BMI	-0.1	-0.2 to 0.1	0.711
	Smoking	2.1	-1.3 to 5.6	0.219
	Hyperlipoproteinemia	2.0	-0.4 to 4.4	0.097
	Diabetes	0.5	-1.5 to 2.6	0.599
	Arterial Hypertension	-0.1	-2.5 to 2.4	0.975
**RVGLS (%)**	Sex	-1.6	-5.1 to 1.9	0.361
	Age	-0.1	-0.2 to 0.1	0.131
	BMI	0.1	-0.3 to 0.4	0.722
	Smoking	4.3	-2.6 to 11.2	0.215
	Hyperlipoproteinemia	1.5	-3.2 to 6.3	0.516
	Diabetes	0.9	-3.2 to 5.0	0.667
	Arterial Hypertension	0.3	-4.9 to 5.4	0.920
**RVGRS (%)**	Sex	3.1	-0.8 to 7.1	0.117
	Age	0.1	-0.1 to 0.3	0.075
	BMI	-0.1	-0.5 to 0.3	0.574
	Smoking	0.5	-7.5 to 8.6	0.894
	Hyperlipoproteinemia	-4.9	-10.4 to 0.6	0.081
	Diabetes	3.2	-1.6 to 8.0	0.181
	Arterial Hypertension	3.1	-2.6 to 8.7	0.278
**RVGCS (%)**	Sex	-1.7	-3.6 to 0.2	0.079
	Age	-0.1	-0.1 to 0.1	0.258
	BMI	0.1	-0.1 to 0.3	0.329
	Smoking	-0.8	-4.7 to 3.1	0.689
	Hyperlipoproteinemia	1.8	-0.8 to 4.5	0.169
	Diabetes	-2.3	-4.6 to -0.1	**0.047**
	Arterial Hypertension	-1.3	-4.0 to 1.4	0.339

Abbreviations: LV = left ventricle, RV = right ventricle, LA = left atrium, RA = right atrium, EF = ejection fraction, EDMi = end-diastolic mass index, EDVi = end-diastolic volume index, ESVi = end-systolic volume index, SVi = stroke volume index, ECV = extracellular volume, GLS = global longitudinal strain, GRS = global radial strain, GCS = global circumferential strain

Supplementary Figure 1: Exemplary balanced steady-state free precession cine-image of a 72-year-old male patient with liver cirrhosis based on primary sclerosing cholangitis (Child-Pugh score B, MELD-score 24 points) during post-processing (Cvi42, Circle Cardiovascular Imaging).

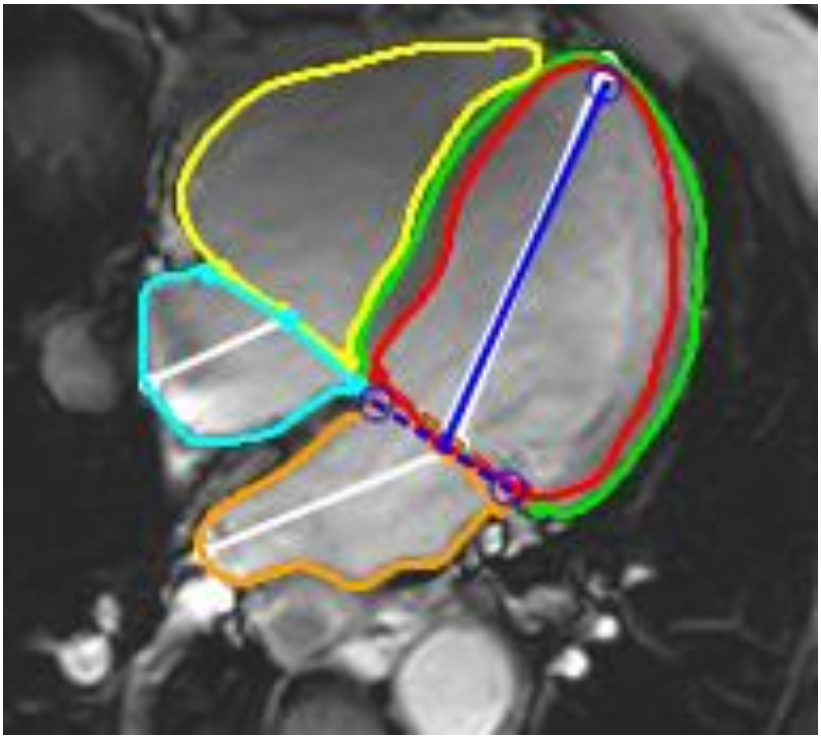

Legend:
The figure displays a 4-chamber view at end-diastole including the endocardial and epicardial contours of the left ventricle (colored in red and green, respectively), as well as the endocardial contours of the right ventricle (yellow), the right atrium (blue), and the left atrium (orange).

References Data Supplement

1 Lang RM, Bierig M, Devereux RB et al (2005) Recommendations for chamber quantification: a report from the American Society of Echocardiography's Guidelines and Standards Committee and the Chamber Quantification Writing Group, developed in conjunction with the European Association of Echocardiography, a branch of the European Society of Cardiology. J Am Soc Echocardiogr 18:1440-1463

2 Russo C, Hahn RT, Jin Z, Homma S, Sacco RL, Di Tullio MR (2010) Comparison of echocardiographic single-plane versus biplane method in the assessment of left atrial volume and validation by real time three-dimensional echocardiography. J Am Soc Echocardiogr 23:954-960
